# Poverty and health – does parenthood matter? Trends in income inequality in self-rated health among parents and non-parents in Germany from 2009 to 2024

**DOI:** 10.1186/s12889-026-27495-x

**Published:** 2026-05-09

**Authors:** Petra Rattay, Florian Beese, Stefanie Sperlich, Nico Dragano, Niels Michalski

**Affiliations:** 1 Department of Epidemiology and Health Monitoring, Robert Koch Institute, Berlin, Germany; 2 Institute of Medical Sociology, Centre for Health and Society, Medical Faculty and University Hospital, Heinrich Heine University Düsseldorf, Düsseldorf, Germany; 3 Department of Medical Sociology, Hannover Medical School, Hanover, Germany

**Keywords:** Poverty, Income, Socioeconomic position, Social inequality, Subjective health, Health inequity, Parenthood, Trend analysis, COVID-19 pandemic

## Abstract

**Background:**

Income inequalities in health are a major public health issue in modern societies. This study investigated an understudied topic by comparing trends in income inequalities in self-rated health (SRH) between parents and non-parents.

**Methods:**

We used data from eight surveys of the GEDA study series (2009–2023) and from the first survey of the RKI Panel (2024), including a total of 106,399 randomly sampled participants aged 25–59. Age-adjusted prevalence of good SRH was calculated for each cross-section stratified by income groups and parental status. Moderation analyses using Poisson regressions with three-way interactions between income, parental status and survey were conducted. Trends in income-related health inequality were analysed based on the Slope Index of Inequality and the Relative Index of Inequality stratified by parental status.

**Results:**

In 2009, 75.7% of women and 77.7% of men reported good SRH. For the total sample, no significant changes in good SRH were observed until the onset of the COVID-19 pandemic, when a notable increase was recorded (women: 80.2%, men: 81.9%). In 2022, the prevalence returned to pre-pandemic levels and reached its lowest point in 2024 (women: 68.1%, men: 71.6%). Non-parents and those at risk of poverty were less likely to report good SRH. The moderation analysis revealed a decline in good SRH among those at risk of poverty, particularly in non-fathers (from 2014/15 onwards) and non-mothers (after 2014/15). A decline in good SRH was also observed among mothers at risk of poverty in 2023/24. These findings were confirmed by the results on absolute and relative income-related inequalities in SRH.

**Conclusions:**

Even before the pandemic, income-related health inequalities in Germany were increasing to the detriment of non-parents at risk of poverty. This could be due to the prolonged strain on this group caused by the ongoing polycrisis. Parenthood may buffer some of the stress associated with poverty, particularly in women. However, selection effects could also be at play, meaning that people in poor health are less likely to become parents or earn higher incomes. To identify health trends early on and promote targeted policy interventions to improve the health of people living in poverty, health inequalities should be regularly monitored for different population groups.

**Supplementary Information:**

The online version contains supplementary material available at 10.1186/s12889-026-27495-x.

## Background

Even in a wealthy country like Germany, poverty is a major problem. Living in (relative) poverty is often associated with marginalisation and exclusion from participation in economic, social and cultural activities [1]. Poverty and social exclusion are also driving forces of health inequalities [2]. Numerous studies have demonstrated that – across countries [3–8] and also in Germany [9–11] – poverty or low income is strongly associated with poor self-rated health (SRH). However, the associations between poverty and health are dynamic and can vary by place, time and population group [8, 12–14]. Continuous monitoring of income inequality in health over time can identify adverse trends. However, in Germany, only a few studies have examined the association between poverty or low income and SRH over time [13, 15–17].

Furthermore, in order to develop targeted policy measures to reduce health inequities, it is crucial to identify the population groups in which income-related health inequalities are most pronounced. However, studies analysing time trends in income-related health inequalities stratified by specific population groups are scarce. In the case of Germany, we only found one study analysing trends in socioeconomic inequality in SRH stratified for migrants and non-migrants [12].

A population group that plays a key role regarding the impact of poverty on health is the family [18]. In 2024, 8.4 million families with minor children lived in Germany [19]. Of these, 13.4% were at risk of poverty [20]. To date, the focus in public health is primarily on the impact of poverty on the health and development of children and adolescents [21, 22], with only little consideration given to the health of parents. There are some studies on the SRH of women and men living with minor children (parents) compared to those living without children (non-parents), showing mixed results: while some studies found better SRH among parents [23, 24], other studies have observed worse SRH [25] or no differences at all between parents and non-parents [26, 27]. However, most of the studies included women only.

These mixed findings can be explained using the framework of the demands-rewards perspective, which states that parenting involves both rewards and pleasure as well as challenges and stress [28]. On the one hand, parenthood can contribute to well-being and good health by providing close, intimate and long lasting social and emotional relationships, mechanisms of structuring everyday life, social control and meaningfulness of life. On the other hand, living with children is associated with numerous demands and obligations, less free time, as well as conflicting role expectations that can lead to psychological distress, dissatisfaction and poor health [28, 29].

Furthermore, parents’ health is largely dependent on contextual factors, as these influence the demands and rewards of parenting [24, 30]. The associations between parenthood and health vary according to gender [31, 32], age [32], the stage in the life course [33], the parenting stage [34], the partner or marital status [35, 36], and the employment status [35, 37]. However, the role of socioeconomic status in parental health has rarely been examined up to now [38]. A study has shown that parents in Germany with low incomes have worse health than those with higher incomes [39], but no comparison has been made with non-parents. Furthermore, the health of single parents is often discussed in relation to poverty, with the assumption that poorer health in this group can be explained by poverty or low income [36, 40, 41]. Especially in demographic research, some studies found that poverty negatively impacts parents’ well-being [42, 43], while some other studies concluded that parenthood leads to lower well-being due to high opportunity costs (in terms of career, income and education), particularly among wealthy and well-educated parents [42, 44]. With regard to SRH, a study from Sweden has confirmed this finding: while motherhood was associated with poor SRH among women in the highest income group, no differences in SRH were found in the low-income group according to parental status [25]. However, a study from France observed no differences in SRH between mothers and non-mothers in the low and high-income groups, whereas differences occurred in the middle-income stratum [23]. At present, there are no studies available for Germany that have analysed the interaction of parental status and income with regard to SRH.

In addition, societal conditions as well as social change influence both the health of parents and the association between poverty and parental health. Nomaguchi and Milkie [24] have stated that historical time is a key context that shapes the levels and types of demands and rewards of parenting. For the US, they reported that mothers living with minor children in the 1990s or earlier were less healthy than women without minor children. By contrast, from 1997 to 2018, mothers were observed to be healthier than non-mothers [24]. However, it has not yet been analysed whether a similar trend can be observed in Germany, or if it also applies to parents at risk of poverty. Thus, there has been insufficient research into how the interplay between parental status and income has developed in recent years.

In the last few decades, Germany has undergone major social changes, such as the transformation of the economy from a conservative to a more liberal labour market [45]. Focusing specifically on the period from 2009 to 2024 in Germany, the global financial crisis of 2008/2009 and the COVID-19 pandemic with its onset in 2020 stand out in particular. In both 2009 and 2020, German gross domestic product (GDP) fell below the previous year’s level [46]. Both were followed by economic slowdowns and financial and social crises. Furthermore, recent years have been marked by multiple crises, including the climate crisis and its consequences, global conflicts with social and economic repercussions, immigration, inflation and recession, housing shortages and rising living costs. McNamara and Bambra [47] refer to these multifaceted, concurrent and synergistic contemporary crises as a ‘global polycrisis’, which is closely linked to health inequalities.

Especially the COVID-19 pandemic put parents under particular stress, impacting them in multiple ways through the containment measures (such as closures of workplaces, schools and daycare centres) as well as the multiplication of parents’ responsibilities and the reorganisation of the family’s everyday life [48, 49]. Families living in poverty were particularly hard hit. Mothers and fathers in a low socioeconomic position complained more often about a drop in income and were also more likely to report problems with childcare than wealthier parents [50]. At the onset of the pandemic, international [51, 52] as well as German [53–56] studies observed a decline in life satisfaction and mental health among parents – particularly among mothers. However, most of these studies focused on mental health problems and distress in the periods at the beginning of the pandemic and did not reflect longer-term health trends.

Currently, there are no studies in Germany investigating whether the association between poverty and SRH varies according to parental status, and whether this association has changed over time.

Against this background, we aim to examine the association between income and SRH among parents compared to non-parents and how it might have changed from 2009 to 2024. Additionally, we intend to quantify the extent of income-related inequality in SRH among parents and non-parents during this time period. On the one hand, it can be assumed that parents living in poverty are particularly burdened by the multiple societal crises and therefore have poorer health than non-parents at risk of poverty. On the other hand, it can also be assumed that parenthood is a social resource that can buffer the stresses of societal crises, even among those at risk of poverty.

In detail, this paper examines the following *research questions*:

- How has good SRH of women and men aged 25 to 59 changed from 2009 to 2024? Does the temporal trend in good SRH differ between women and men with low (at risk of poverty), middle and high incomes and between parents and non-parents?
- Does the association between income and good SRH vary over time between parents and non-parents?
- Does income-related inequality in good SRH vary over time between parents and non-parents?

## Materials and methods

### Data

This study used data from nationwide population-based surveys conducted by the Robert Koch Institute (RKI) as part of its health monitoring programme on behalf of the Federal Ministry of Health. The *German Health Update (GEDA)* comprises a series of cross-sectional surveys (GEDA 2009, GEDA 2010, GEDA 2012, GEDA 2014/2015-EHIS, GEDA 2019/2020, GEDA 2021, GEDA 2022, GEDA 2023) conducted at irregular intervals but with similar content and drawn from independent samples. We also included the first annual survey wave of the panel study *Health in Germany* (RKI Panel 2024). All surveys provide representative data for the German-speaking population of Germany aged 18 years and older residing in private households for the respective survey periods.

All surveys of the GEDA study series – with the exception of GEDA 2014/2015-EHIS – were conducted as a telephone survey using a fully structured questionnaire (Computer Assisted Telephone Interview, CATI). Participants were selected using a random sample of landline and mobile phone numbers (dual-frame method) [57]. In GEDA 2014/2015-EHIS and in the RKI Panel 2024, random samples were drawn from the population registers of the residents’ registration offices instead of telephone samples. In addition, the survey mode changed from telephone interview to web-based or written questionnaire [57]. Each survey was approved by the Federal Commissioner for Data Protection and Freedom of Information, and verbal or written informed consent was obtained in advance from all participants [57]. In the GEDA studies, the response rate (proportion of completed interviews to the total sample, adjusted for neutral non-respondents) ranged from 16.1% to 29.1%. For the RKI Panel 2024, the response rate was 22.4%. A detailed description of the methodology of the GEDA studies and the RKI Panel 2024 can be found in Allen et al. [58] and Lemcke et al. [59].

Across all included surveys, a total of 113,429 women and 95,427 men participated. For this analysis, the sample was limited to participants aged 25 to 59 years, as the probability of living with one’s own minor children is significantly lower at younger and older ages. This results in a sample size of 58,102 women and 48,297 men aged 25 to 59 years (weighted proportions: women: 49.4%, men: 50.6%). Of these, 23,732 were mothers and 17,008 fathers. Detailed information on the sample sizes for the individual surveys can be found in Table 1.

**Table 1:** Description of the sample

	GEDA09	GEDA10	GEDA12	GEDA14/15-EHIS	GEDA19/20-EHIS (before 15/03/20)	GEDA19/20-EHIS (from 15/03/20) / GEDA21	GEDA22	GEDA23	PANEL24	Total
Survey period	07/08 − 05/09	09/09 − 07/10	03/12 − 02/13	11/14 − 07/15	04/19–14/03/20	15/03/20 − 09/20, 06/21 − 12/21	02/22 − 01/23	01/23 − 02/24	05/24 − 01/25	07/08–01/25
	*n (%)* ^*a*^	*n (%)* ^*a*^	*n (%)* ^*a*^	*n (%)* ^*a*^	*n (%)* ^*a*^	*n (%)* ^*a*^	*n (%)* ^*a*^	*n (%)* ^*a*^	*n (%)* ^*a*^	*n (%)* ^*a*^
Total	13,389	13,938	10,419	14,093	6,769	7,672	14,065	12,153	13,901	106,399
Self-rated health										
Very good / good	10,616 (76.7)	11,197 (77.9)	8,190 (76.1)	10,793 (75.1)	5,390 (74.5)	6,501 (81.0)	11,063 (74.9)	9,413 (73.2)	10,252 (69.9)	83,415 (75.2)
Fair / bad / very bad	2,760 (23.3)	2,728 (22.1)	2,224 (23.9)	3,261 (24.9)	1,377 (25.5)	1,169 (19.0)	2,996 (25.1)	2,733 (26.8)	3,637 (30.1)	22,885 (24.8)
Missing	13	13	5	39	2	2	6	7	12	99
Gender										
Female	7,771 (49.3)	8,087 (49.4)	5,232 (49.3)	7,941 (49.5)	3,436 (49.7)	4,017 (49.2)	7,435 (49.6)	6,355 (49.6)	7,828 (49.3)	58,102 (49.4)
Male	5,618 (50.7)	5,851 (50.6)	5,187 (50.7)	6,152 (50.5)	3,333 (50.3)	3,655 (50.8)	6,630 (50.4)	5,798 (50.4)	6,073 (50.7)	48,297 (50.6)
Age group										
25–29	1,367 (12.3)	1,451 (11.6)	920 (11.1)	1,560 (13.0)	479 (10.2)	486 (11.3)	937 (9.1)	884 (9.7)	1,665 (13.0)	9,749 (11.2)
30–34	1,436 (11.5)	1,664 (12.4)	1,228 (13.6)	1,576 (12.6)	760 (15.0)	731 (14.2)	1,355 (13.0)	1,183 (12.7)	1,851 (14.1)	11,784 (13.1)
35–39	1,958 (14.9)	1,900 (13.7)	1,014 (11.9)	1,820 (12.2)	802 (13.6)	896 (14.6)	1,660 (15.5)	1,425 (15.4)	2,023 (14.5)	13,498 (14.1)
40–44	2,582 (17.6)	2,532 (17.6)	1,972 (15.8)	1,929 (12.9)	860 (13.4)	997 (13.0)	1,915 (14.0)	1,702 (13.8)	1,855 (13.9)	16,344 (14.7)
45–49	2,342 (16.6)	2,460 (16.8)	1,693 (17.8)	2,559 (17.1)	915 (12.3)	1,021 (11.3)	1,968 (13.7)	1,757 (13.9)	1,435 (12.7)	16,150 (14.8)
50–54	1,874 (14.1)	2,075 (14.7)	2,153 (16.0)	2,708 (18.0)	1,365 (17.6)	1,594 (18.9)	2,572 (14.3)	2,073 (14.0)	2,250 (14.2)	18,664 (15.5)
55–59	1,830 (13.1)	1,856 (13.1)	1,439 (13.8)	1,941 (14.1)	1,588 (17.9)	1,947 (16.8)	3,658 (20.4)	3,129 (20.5)	2,822 (17.6)	20,210 (16.6)
Income										
<60% (at risk of poverty)	1,296 (12.6)	1,428 (13.5)	1,033 (13.3)	1,859 (14.5)	738 (17.7)	736 (16.7)	1,289 (15.2)	1,170 (16.3)	1,544 (14.7)	11,093 (14.8)
60%-<150%	8,566 (67.1)	8,567 (64.6)	5,941 (59.5)	8,421 (61.5)	3,799 (59.0)	4,087 (57.5)	7,766 (59.3)	6,878 (59.6)	7,710 (56.8)	61,735 (60.6)
>=150%	3,527 (20.3)	3,943 (21.9)	3,445 (27.3)	3,813 (24.1)	2,232 (23.2)	2,849 (25.8)	5,010 (25.5)	4,105 (24.0)	4,647 (28.5)	33,571 (24.6)
Parental status										
Non-parent	7,605 (56.2)	7,933 (56.6)	6,241 (57.4)	9,088 (66.2)	4,320 (69.1)	4,830 (66.6)	8,831 (66.1)	7,520 (65.2)	8,153 (63.8)	64,521 (62.9)
Parent	5,756 (43.8)	5,974 (43.4)	4,161 (42.6)	4,941 (33.8)	2,428 (30.9)	2,817 (33.4)	5,202 (33.9)	4,587 (34.8)	4,874 (36.2)	40,740 (37.1)
Missings	28	31	17	64	21	25	32	46	874	1,138
Partner status										
No partner	3,990 (22.8)	4,295 (23.5)	3,690 (28.8)	3,691 (28.1)	2,215 (46.3)	2,424 (42.4)	4,377 (40.1)	3,914 (40.9)	4,450 (36.7)	33,046 (34.1)
Partner	9,236 (77.2)	9,499 (76.5)	6,683 (71.2)	9,941 (71.9)	4,418 (53.7)	5,146 (57.6)	9,475 (59.9)	8,087 (59.1)	9,413 (63.3)	71,898 (65.9)
Missings	163	144	46	461	136	102	213	152	38	1,455
Education										
Low	2,536 (29.8)	2,543 (28.7)	1,478 (24.8)	2,096 (22.0)	748 (21.0)	731 (19.8)	1,199 (18.0)	1,001 (18.7)	1,325 (23.8)	13,657 (22.8)
Middle	6,965 (53.0)	7,092 (52.7)	5,570 (54.5)	8,118 (58.9)	3,289 (57.1)	3,570 (57.0)	6,459 (57.1)	5,590 (57.0)	6,996 (50.7)	53,649 (55.3)
High	3,860 (17.1)	4,285 (18.5)	3,352 (20.7)	3,855 (19.1)	2,711 (21.9)	3,341 (23.2)	6,350 (24.9)	5,521 (24.3)	5,576 (25.5)	38,851 (21.9)
Missings	28	18	19	24	21	30	57	41	4	242
Employment status										
Full-time employed	7,359 (56.0)	7,694 (56.7)	6,286 (56.6)	8,425 (61.4)	4,211 (58.6)	4,837 (59.6)	8,613 (58.5)	7,424 (58.6)	7,660 (60.2)	62,509 (58.5)
Part-time employed	3,087 (20.1)	3,383 (20.9)	2,263 (21.9)	2,902 (18.4)	1,456 (19.4)	1,677 (18.3)	3,361 (21.0)	2,919 (21.2)	3,434 (23.5)	24,482 (20.7)
Non-employed / Others	2,931 (23.9)	2,848 (22.4)	1,869 (21.5)	2,695 (20.2)	1,082 (22.0)	1,133 (22.2)	2,051 (20.5)	1,770 (20.3)	1,868 (16.3)	18,247 (20.8)
Missings	12	13	1	71	20	25	40	40	939	1,161

^*a*^n unweighted; % weighted

### Variables

#### Outcome variable

Self-rated health (SRH) is a valid universal health indicator and a good predictor of health, morbidity and mortality, as well as the use of health services [60, 61]. It is a core variable of the Minimum European Health Module (MEHM 1) [62, 63]. The SRH status was collected using the question “How is your health in general?” The five response categories “very good”, “good”, “fair”, “bad” or “very bad” were grouped into “good SRH” (very good or good) versus “not good SRH” (fair to very bad). Below, we present the results for good SRH.

#### Time variable

Although the nine surveys do not each cover an entire calendar year, we analysed each survey in its entirety, with the exception of GEDA-EHIS 19/20 and GEDA 2021. Here, we combined the two surveys and then divided them into pre- and peri-pandemic observation periods (with a cut point of 15 March 2020, when containment measures began in Germany). The exact survey periods can be found in Table 1.

#### Predictor variables

##### Parental status

The parental status was based on information provided by the study participants on all persons living in the household. We defined women and men as parents when they live together with at least one own child under 18 years. The parental status distinguished between “parent” (minor child(ren) living in the household) and “non-parent” (no minor child(ren) in the household). No differentiation was made between biological children, stepchildren, or adopted children (social parenthood).

##### Poverty

To measure the association between poverty and SRH, we compared different income levels. This also allowed for a more detailed analysis of the extent of health inequalities. The operationalisation of poverty is based on the official EU definition and follows the concept of relative poverty [1]. A person is considered to be at risk of poverty according to the EU definition if he or she has less than 60% of the average income (median) of the national population at their disposal [1]. The disposable household equivalised income comprises the household’s total income after taxes and social transfers divided by the sum of the weights of all household members according to the new OECD scale [64]. The income variable includes imputed values generated through stochastic regression imputation [65]. The median was calculated separately for the total population of each survey; the age restriction to participants aged 25 to 59 years was applied after the median was calculated. For the analyses, the disposable household equivalised income was categorised as “low” (< 60% of the median = at risk of poverty), “medium” (60%–<150% of the median), and “high” (≥ 150% of the median).

#### Control and stratification variables

The following control variables were included: the ages of the respondents were grouped into five-year categories. The partner status was defined by living with a partner in a household (“yes”/“no”) regardless of marital status and gender. The employment status was self-defined and distinguished between “employed full-time”, “employed part-time”, and “not employed” (including unemployed persons, students, pensioners, volunteers, homemakers and others). With regard to the respondent’s educational level, the Comparative Analysis of Social Mobility in Industrial Nations (CASMIN) classification was used (“low”, “medium”, “high”) [66]. In supplementary sensitivity analyses exclusively for parents, the number of children and the age of the youngest child (“0–6 years”, “7–13 years”, “14–17 years”) were also controlled for (see appendix, Fig. A-1).

All analyses were stratified by gender (“women”/“men”).

### Data analysis

In the first step, we provided age-adjusted prevalence for SRH. Age adjustment was achieved by obtaining predictions from averaging the predicted values from Poisson regression models with a log link and age groups as predictor. Estimates were presented graphically stratified by gender, parental status and income for each survey.

In the second step, we deployed Poisson regression models with a log link separately for women and men. These models included age and partner status (model 1) and successively added level of education and employment status (model 2). Both models included three-way interaction terms between survey, parental status and income groups to allow for varying effects of parental status, income and their interaction over time. For easier interpretation of the differences between groups, we derived predictive margins from the fitted models [67]. The predicted probabilities for model 1 were presented graphically. The exact values of the predicted probabilities from model 1 and model 2 were displayed in tables in the appendix. A supplementary sensitivity analysis revealed that the results of model 1 for parents hardly changed when adjusting for the number of the children and the age of the youngest child (see Appendix, Fig. A-1). Therefore, adjusting for these variables did not seem reasonable.

In the third step, we aimed to investigate the income-related inequality in SRH over time by gender and parental status. Particularly, when analysing the temporal dynamics of health inequalities, it is recommended to examine both absolute and relative inequalities, because relative inequalities may increase while absolute inequalities decrease as the prevalence of poor health declines [68, 69]. Therefore, we presented model-based inequality measures, namely the Slope Index of Inequality (SII) and the Relative Index of Inequality (RII). These indices summarise absolute (SII) and relative (RII) differences in one estimate while accounting for the distribution of income groups and the overall outcome prevalence, allowing comparability across surveys [70]. For this purpose, we first transformed the categorical income variable into a numeric cumulative rank variable (ridit score) following Bross’s method [71], which assigns each income category to the midpoint of its cumulative population proportion. We then used these scores as the independent variable in regression models predicting SRH: SII was estimated using models with an identity link and Gaussian distribution producing the absolute prevalence difference between the extremes of the income distribution; RII was estimated using models with a log link and Poisson distribution, producing the relative prevalence ratio between these extremes. Models were adjusted for age and partner status mirroring the covariate set of model 1 of the second step. The exact values ​​of the SII and RII were presented in the tables in the appendix.

Cases with missing data on variables other than income, for which missing values were imputed (see above), were excluded via listwise deletion (see Table 1).

For all estimators we reported 95% confidence intervals or p-values. Since no data are available between 07/2015 and 04/2019, this period was shown in all figures using a dashed line.

#### Weighting

In order to adjust for deviations in the survey samples from the underlying target population due to sampling design and selective non-response, a weighting factor was applied in the analysis of each survey. These weighting factors adjust the survey samples to the population structure of Germany with regard to sex, age, federal state and education. Starting from GEDA 2014/2015-EHIS, the regional settlement structure (district or municipality) was also taken into account. Regarding the RKI Panel 2024, the weighting also included an adjustment for single-person versus multi-person households. In addition, a correction factor based on the information in the recruitment study for the RKI Panel 2024 was taken into account to compensate for non-participation in the first annual wave of the repeated panel survey [57].

All analyses were performed with the statistical software R version 4.3.0 [72]. The sample description is given in Table 1.

## Results

### Parental status, income and the associations with good SRH

Among the sample group aged 25 to 59, 41.4% of women and 32.8% of men were parents (see Table A-1 in the appendix). 15.9% of women and 13.8% of men were at risk of poverty, while 22.0% of women and 27.1% of men were in the high-income group. Taking parental status into account, 16.1% of mothers, 15.3% of non-mothers, 11.2% of fathers and 14.8% of non-fathers were at risk of poverty. It is striking that, among women, mothers and, among men, non-fathers were more often at risk of poverty. On the other hand, non-mothers (26.4%) and non-fathers (30.3%) had high incomes more often than mothers (15.8%) and fathers (20.5%) (see Table A-2 in the appendix).

On average across all surveys, 73.1% of women and 75.6% of men reported good SRH (see Table 2). Parents were more likely to rate their health as good than non-parents. Furthermore, a clear income gradient was evident: while women and men in the highest income group were the most likely to report good SRH, those at risk of poverty were the least likely. Prevalence rates for the middle-income group were intermediate.

**Table 2:** Good SRH among women and men, stratified by parental status and income groups (age-adjusted prevalence of good/very good SRH as %, 95% CI)

	Women	Men
	% (95% CI)	% (95% CI)
Total	73.1 (72.5–73.6)	75.6 (75.0–76.1)
Parental status		
Non-parent	68.8 (68.1–69.5)	72.6 (71.9–73.3)
Parent	79.7 (79.0–80.5)	82.0 (81.2–82.9)
Income		
<60%	57.8 (56.1–59.4)	59.1 (57.2–61.1)
60%-<150%	74.0 (73.4–74.6)	75.7 (75.0–76.4)
>=150%	81.8 (81.0–82.7)	84.3 (83.5–85.1)

### Trends in good SRH stratified by parental status and by income group

**Fig. 1:**
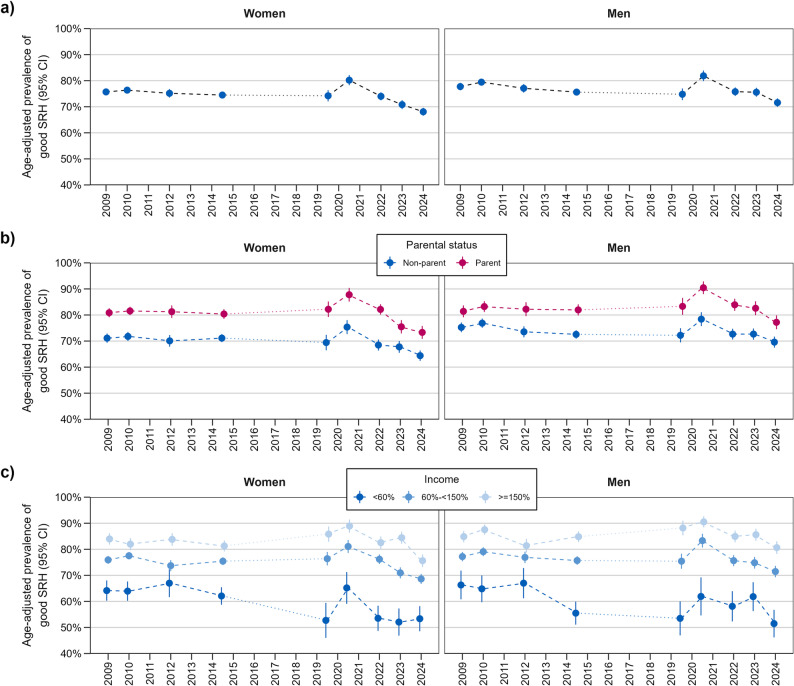
Trends in good SRH among women and men (total (**a**), stratified by parental status (**b**) and income groups (**c**); age-adjusted prevalence rates of good/very good SRH as %, 95% CI). Legend: Age-adjusted prevalence rates from repeated cross-sectional surveys. Each point represents a separate sample; lines indicate trends over time. Tables A-3, A-4 and A-5 in the Appendix present all values

Examining the prevalence of good SRH over time (see Fig. 1a) revealed no profound changes over time until the onset of the pandemic (2020/21), when a notable increase was recorded. In 2022, the prevalence fell back again to the pre-pandemic level. The lowest prevalence over the entire period was found in 2024.

There were no differences in the trend patterns among women and men according to parental status (see Fig. 1b). The prevalence rates were consistently higher among parents than non-parents in each survey. However, differences in prevalence by parental status in 2009 and 2010 were less pronounced for men than for women.

In each survey, a significant income gradient was observed among women and men, to the detriment of those at risk of poverty (see Fig. 1c). However, one exception was found: in 2012, the 95% CI of the low- and middle-income groups overlapped among women, as did the 95% CI of the middle- and high-income groups among men. Notably, a decline in good SRH among those at risk of poverty was evident even before the pandemic, among men in 2014/15 and among women in 2019/20.

### Trends in good SRH among women and men by income, stratified by parental status

**Fig. 2:**
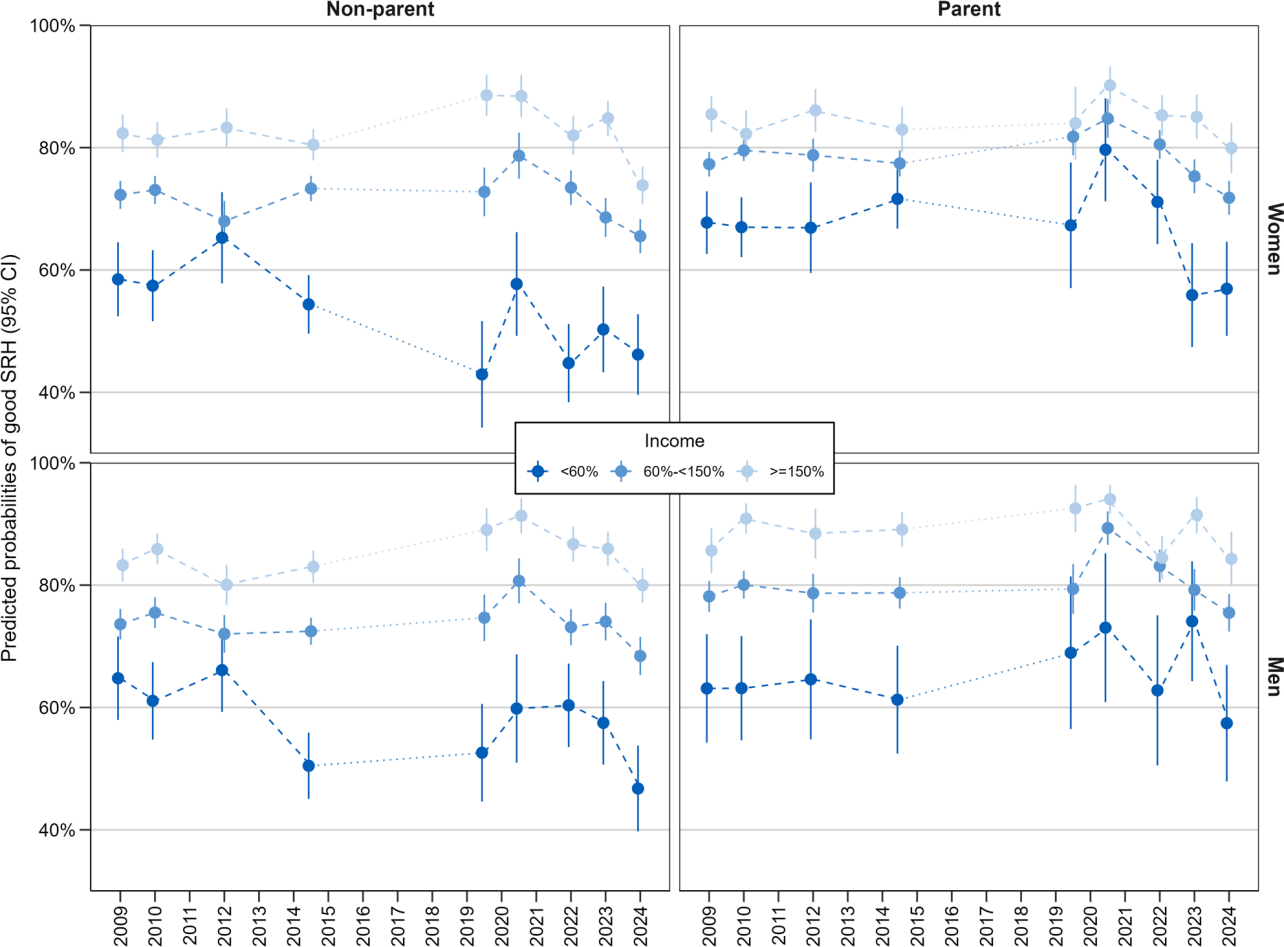
Trends in good SRH among women and men by income, stratified by parental status (predicted probabilities of good/very good SRH as %, 95% CI; adjusted for age groups and partner status). Legend: Predicted probabilities from repeated cross-sectional surveys. Each point represents a separate sample; lines indicate trends over time. Table A-6 in the Appendix presents all values

Figure 2 illustrates the trends in good SRH for the three income groups, stratified by parental status.

Among women, it is striking that income differences were much smaller among mothers than among non-mothers. The decline in prevalence before the onset of the pandemic in 2019/20 among women at risk of poverty was particularly noticeable among non-mothers. Between 2014/15 and 2022, the prevalence of good SRH in the low-income group was significantly higher among mothers than non-mothers. At the beginning of the pandemic, an increase in good SRH was observed, particularly among women at risk of poverty. This was evident for both mothers and non-mothers. Among mothers, at this time (2020/21) no significant differences in SRH between the income groups were observed. However, in 2023/24, low-income mothers rated their health significantly less often as good than in previous years. Comparing middle- and high-income mothers, the differences in the prevalence of good SRH were relatively small. In some surveys, no significant differences were evident at all.

Among men, differences between fathers and non-fathers were less pronounced. However, a similar pattern was found among men as among women in the at-risk-of-poverty group: a decline in the prevalence of good SRH was observed before the pandemic (in 2014/15 and 2019/20) only among non-fathers. In this group, prevalence rose slightly during the pandemic, but then declined again in 2024. In the high- and middle-income groups, similar trends were observed among men for both fathers and non-fathers. Overall, the prevalence tended to be somewhat higher for fathers than for non-fathers in both income groups.

When adjusting additionally for employment status and education (see Table A-6 in the appendix), significant differences in good SRH by income group were found for mothers only in 2023 and 2024 and for fathers in 2014/15 and 2023. Among non-mothers, the adjusted prevalence of good SRH was significantly lower in the at-risk-of-poverty group than in the middle- and high-income groups in almost all surveys. For non-fathers, differences in good SRH by income were evident from 2014/15 to 2024 (with the exception of 2020/21), but not in the form of a gradient across all three income groups.

### Trends in absolute and relative income-related health inequalities among parents and non-parents

**Fig. 3:**
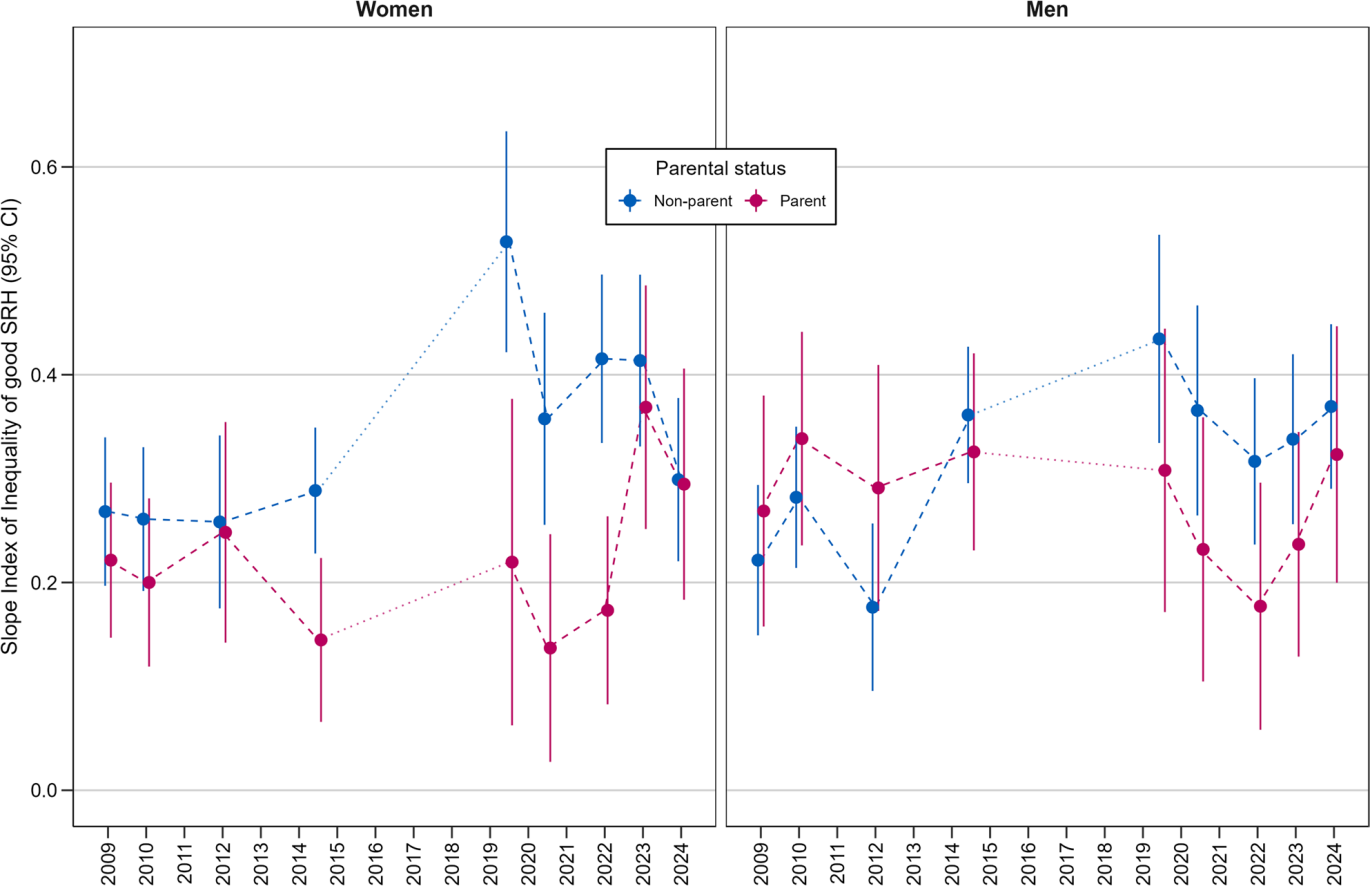
Trends in absolute income inequalities in good (good/very good) SRH among parents and non-parents (Slope Index of Inequality (SII), 95% CI; adjusted for age groups and partner status). Legend: SII from repeated cross-sectional surveys. Each point represents a separate sample; lines indicate trends over time. Interpretation of SII: An SII of 0.53 (see GEDA 2019/20; non-mothers) indicates a 53-percentage-point difference in the proportion of the population (non-mothers) with good SRH between the lowest and highest income groups. Table A-7 in the Appendix presents all SII values

**Fig. 4:**
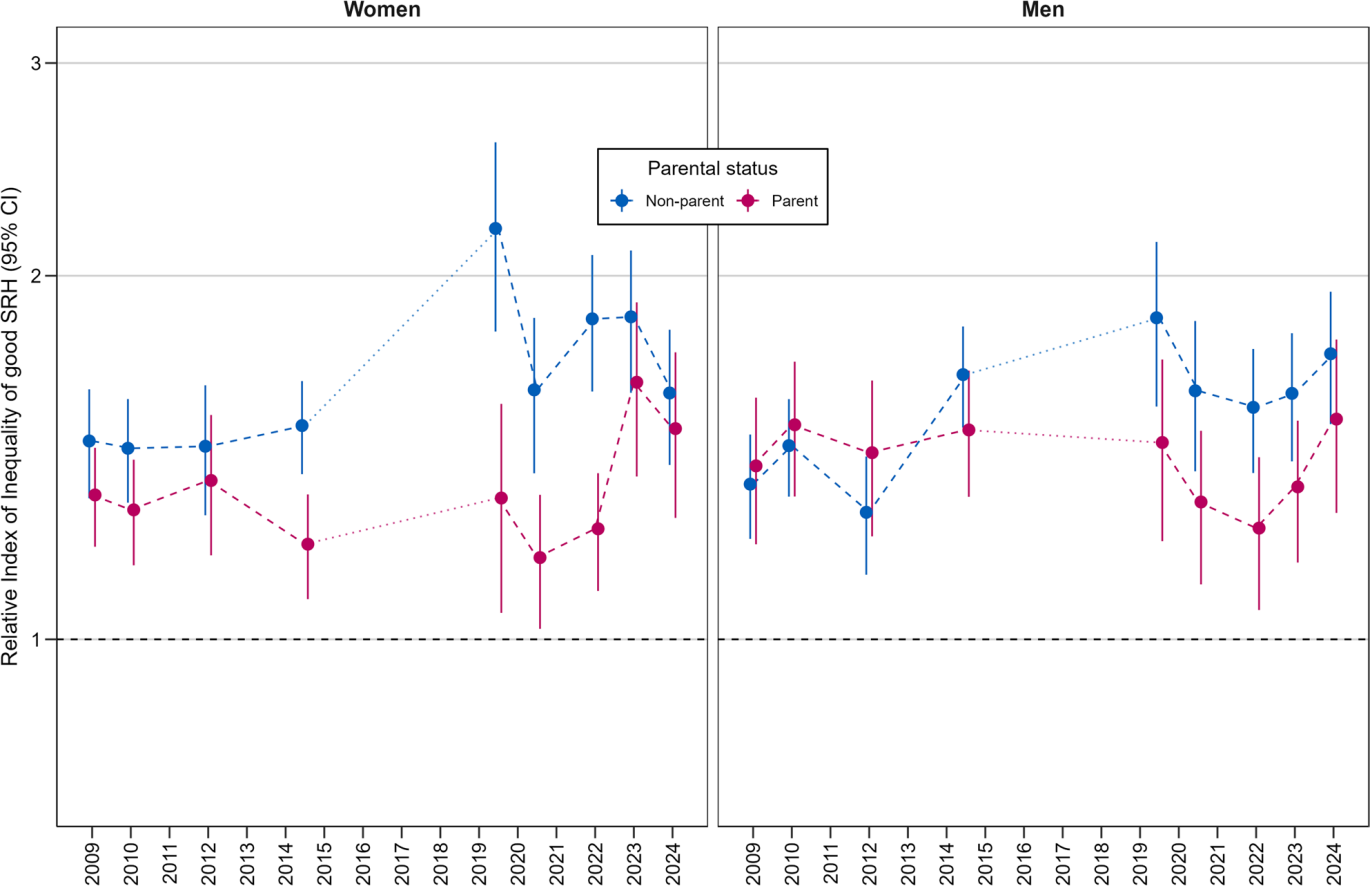
Trends in relative income inequalities in good (good/very good) SRH among parents and non-parents (Relative Index of Inequality (RII), 95% CI; adjusted for age groups and partner status). Legend: RII from repeated cross-sectional surveys. Each point represents a separate sample; lines indicate trends over time. Interpretation of RII: An RII of 2.18 (see GEDA 2019/20; non-mothers) indicates that people (non-mothers) with the highest incomes are 2.18 times more likely to report good SRH than people (non-mothers) with the lowest incomes. Table A-7 in the Appendix presents all RII values

Figures 3 and 4 illustrate the absolute (SII) and relative (RII) income inequalities in SRH among parents and non-parents. Significant absolute and relative income inequalities in SRH were evident in all surveys for both parents and non-parents of both genders.

Among women, the trend patterns of the SII and RII were very similar. Different inequality patterns were evident in both the SII and RII depending on parental status. In the period between 2014/15 and 2022, the SII as well as the RII for non-mothers were significantly higher than for mothers. This was particularly evident in the pre-pandemic period of 2019/20, when a sharp increase in income inequality among non-mothers was observed. In 2023, there was a noticeable increase in the SII and the RII among mothers. In 2024, the SII and RII of mothers and non-mothers were almost at the same level.

Among men, the patterns of absolute and relative inequality were similar for men with and without children. The 95% CIs for both the SII and RII overlapped for fathers and non-fathers in all surveys. However, the SII and the RII tended to be lower for non-fathers than for fathers between 2009 and 2012, but higher from 2014/15 onwards.

In sum, absolute and relative income-related SRH inequality were both quite high in the period before the pandemic (2019/20), particularly among non-mothers and, to a lesser extent, among non-fathers. Furthermore, income-related SRH inequality declined considerably at the beginning of the pandemic (2020/21), regardless of parental status or gender. Among men, inequality subsequently rose again towards the end of the pandemic and afterwards. Among women, an increase occurred in 2022, followed by a slight decline in 2024. Overall, the patterns of absolute and relative income inequality in SRH were very similar within each of the four parental status subgroups.

## Discussion

### Summary of the results

The present analysis examined the trend in income inequality in good SRH from 2009 to 2024 in Germany and how this trend differed between parents and non-parents.

Comparing the prevalence of good SRH in 2024 with that of 2009, a significant decrease was observed among parents and non-parents and across almost all income groups, but particularly among women and men at risk of poverty. A closer look at the trend of good SRH revealed only little variation from 2009 until the start of the pandemic. At the onset of the pandemic (2020/21), the prevalence of good SRH increased but subsequently declined, reaching its lowest level in 2024. Throughout the entire period, parents consistently reported good SRH more frequently than non-parents. This was true for both genders. Furthermore, a clear and persistent income gradient was evident in each survey, with the lowest prevalence of good SRH observed among women and men at risk of poverty.

Examining whether the association between income and good SRH varied over time between parents and non-parents revealed differences. Particularly among those at risk of poverty, a significant decline in good SRH was observed among non-fathers from 2014/15 onwards, and among non-mothers after 2014/15. In 2023 and 2024, there was also a marked decline in good SRH among mothers. Absolute and relative income-related health inequalities peaked in the years before the pandemic, narrowed at its onset, and then widened again in subsequent years. This was particularly evident among non-mothers, but also among non-fathers and to a lesser extent among mothers.

### Contextualisation of the findings and comparison with the current state of research

Regarding the overall trend, the most notable finding is that good SRH became more prevalent especially at the beginning of the pandemic. This applied to people aged 25 to 59, regardless of gender, parental status or income group. This is remarkable because one might have expected that the widely reported increased psychosocial stress caused by the pandemic’s challenges and containment measures [48–50] would lead to poorer health – at least among certain population groups such as parents struggling to balance family and work. However, numerous studies have also observed an improvement in SRH, particularly at the beginning of the pandemic [11, 73–76]. There are various explanations for this finding: first, the positive impact of perceived greater solidarity and stronger social cohesion at the beginning of the pandemic on the general health assessment is discussed [11, 77]. Furthermore, it is hypothesised that containment measures such as working from home, reduced working hours, and having more time for private life and leisure led to a slower pace of work and life. This may have reduced stress levels for certain population groups in the short term [74, 76, 78], and this is perhaps reflected in better SRH. However, it is also assumed that the improvement in health is not attributed to an actual increase in health, but rather to a different health assessment. It is supposed that people compared their health with that of those affected by or at risk of contracting the coronavirus, and thus partly rated their own health more positively [74–76]. Hence, it would be helpful to compare our results on SRH with more objective health measures. Nevertheless, medical diagnoses may also be influenced by the pandemic, for example due to lower utilisation of physicians or more limited healthcare provision [79].

However, this improvement in SRH at the beginning of the pandemic only lasted for a short time. In 2022, the prevalence of good SRH had fallen back to the pre-pandemic levels. After the pandemic, a further decline in the prevalence of good SRH was observed in 2023 and especially in 2024. This could be related to ongoing and emerging crises. It remains to be seen whether this is a longer-term trend in declining health or a short-term period effect.

With regard to *parental status*, it was observed in all surveys that parents rated their health as better than non-parents. For the US, Nomaguchi and Milkie [24] found also that, between 1997 and 2018, mothers aged 18 to 59 were more likely to rate their health as good than non-mothers. Our results thus support their ’parenthood gap in health‘ hypothesis [24]. However, the study by Nomaguchi and Milkie only included women. To our knowledge, no trend analysis is currently available that compares the SRH of fathers and non-fathers. Nor are there any trend analyses on SRH of parents and non-parents for Germany.

Referring to the demands-rewards model [28], it can be assumed that the benefits of parenthood – such as forming close social and emotional relationships with children, having more social contact within the child’s environment (e.g. childcare, school, neighbourhood and other families) and having a more structured daily routine – outweigh such disadvantages as obligations, strain and distress. In addition, social control may positively influence the health of parents, who focus more on their health in order to be good caregivers and role models for their children [24]. Even during the pandemic, the positive effects of parenthood seem to have outweighed the disadvantages for many parents, which were exacerbated in some cases by containment measures (e.g. the closure of childcare services and schools). This argumentation is based on the assumption that the rewards and benefits associated with parenthood affect health (‘causality’ hypothesis). However, it is also possible that healthy women and men are more likely to enter into a partnership and have children than those with severe chronic illnesses or mental health problems (‘selection’ hypothesis) [25, 80]. Nevertheless, studies on SRH and well-being tend to support the causality hypothesis more strongly [42, 80].

With regard to *income*, a steep gradient in SRH was observed across all surveys. From 2009 to 2024, both women and men at risk of poverty were less likely to report good health than those in the high-income and middle-income groups. This finding is supported by other trend analyses for Germany and internationally [4, 6, 17].

Notably, the prevalence of good SRH had already begun to decline significantly among the at-risk-of-poverty group prior to the pandemic (among men from 2014/15 onwards, among women after 2014/15), although this trend was not observed among the middle- and high-income groups. Data from the German Socio-Economic Panel also indicated a temporary deterioration in SRH among the low-income group in 2018, while this was not observed among the middle- and high-income groups [17]. Groh-Samberg et al. also found that health satisfaction declined only among the poor and those in precarious living situations when comparing the periods 2003/07 and 2013/17 [81]. However, as these two trend analyses relate to the entire adult population, they are only partially comparable with our sample. Pförtner and Demirer [74] observed a slight increase in poor SRH among the working poor compared to non-working poor individuals in the post-recession phase (from 2010 until the beginning of the pandemic). Similarly, data from the UK on the trend in SRH among people under 55 confirm that, even before the pandemic (in 2017/18), a significant deterioration in SRH was observed among people in persistent poverty [4]. In Belgium, a significant decline in SRH was also found among people aged 25 to 59 between 2008 and 2018 in the lowest income quintile [6]. In both countries this was not the case for people not living in poverty [4, 6].

Although the results of the studies are broadly similar, they do not offer clear explanations for this trend. A conceivable rise in absolute poverty within the risk-of-poverty group, which might be expected to worsen health, is not supported by other studies, which show that the disposable household income gap for the first decile did not decrease but instead remained stagnant at the 1999 level until 2022 [82]. Rather, the gap between poor and higher-income households has widened significantly in Germany since 1999, driven by stronger income growth in the upper deciles [82]. Therefore, only relative income inequalities could explain the detrimental health effects observed among lower-income groups, as found in a few studies [83]. A further explanation could be that the financial crisis of 2008/09 did not have an immediate impact on health, only becoming apparent after a certain time lag. Avdic et al. [84] demonstrated that a decline in mental health was observed in Germany several years after the 2008/09 financial crisis. They concluded that economic crises have long-term negative effects on mental health that persist even after the economy has recovered [84]. It can be hypothesised that the mechanisms are similar in SRH.

From 2022 onwards, the prevalence of good SRH was significantly lower among women at risk of poverty than in 2009. This trend was less evident among men at risk of poverty. Nevertheless, in 2024, the prevalence of good SRH was lower than in 2009 for all three income groups among both genders. Contrary to our results, the SOEP data showed for Germany hardly any changes in the prevalence of good SRH within income groups when comparing 2008 or 2010 with 2022 [17]. However, the years 2023 and 2024 are not included in their analysis. In the UK [4], the trend of poor SRH between 2014 and 2023 is similar among those in poverty and those not in poverty, but with a significant increase in poor SRH for both income groups. However, as mentioned above, there was a temporary increase in poor SRH among those in poverty in 2017/18 [4]. Further studies are required that analyse the association between social macro-indicators, including the economic situation, and health inequalities.

The focus of our analysis is on the *interaction between income and parental status* on SRH in trend. The key finding is that the decline in the prevalence of good SRH among men at risk of poverty from 2014/15 and among women at risk of poverty from 2019/20 onwards is particularly evident among non-parents. This also applies to the results for absolute and relative income inequality in SRH.

However, gender differences are evident: among fathers, health inequality remained roughly the same across all surveys. By contrast, in women income inequality in SRH was lower among mothers than among non-mothers between 2014/15 and 2022. During this period, the differences in the prevalence of the three income groups were smaller and income-related inequality was lower among mothers than among non-mothers. At the beginning of the pandemic, there were no differences at all between the three income groups among mothers, due to an improvement in SRH among mothers at risk of poverty. Thus, it was particularly mothers at risk of poverty who seemed to be more resilient in the face of the heightened distress often caused by school and daycare closures, as well as to the negative health effects. During the pandemic, when contact with people outside the household was restricted, many mothers presumably viewed the increased time spent with their families positively, regardless of their income.

In line with ‘role enhancement theory’ [85, 86], these findings can be interpreted to mean that low-income mothers, who often have limited job and career opportunities, may experience recognition and appreciation through their mother role, which can have a positive impact on their health. Among non-mothers, however, potential stress resulting from poverty cannot be offset by social approval in the family sphere. In contrast to our findings, a Swedish study with data from 1996 to 2003 found no differences in SRH by mother status in the low- and middle-income groups. On the other hand, in the high-income group mothers were more likely to report poor SRH than non-mothers [25]. In line with ‘role strain theory’ [86, 87], this was attributed to high levels of stress and difficulties in reconciling family and work. As our study used more recent data and was based on household income and not individual income, the results cannot be compared. However, the decline in SRH among mothers at risk of poverty, and thus the increase in income-related health inequality, that we observed in 2023 and 2024, could also be interpreted in terms of ‘role strain theory’ as an overload for poor mothers after years of pandemic, as well as ongoing and new crises such as the war in Ukraine, the sharp price increases for energy and food due to high inflation in 2022 and 2023 [88] and a housing shortage. In addition, selection effects must be assumed, as long-term and serious illnesses in women can influence both the ability to find well-paid employment and to start a family [80, 89, 90]. Longitudinal studies are required to clarify the direction of the associations between parenthood, income and health.

In addition, we explored how adjusting for educational level and employment status affects the associations between income and SRH among parents and non-parents. We found a noticeable weakening of the associations. This means, that some of the differences in SRH by income and parental status might be attributable to education-related differences such as knowledge or health literacy. Since education is associated with health behaviour [91], income disparities in SRH may also be attributed to differences in health behaviour. Similarly, health behaviour could explain differences in SRH by parental status. Parents, especially mothers, tend to consume less tobacco and alcohol and more fruits and vegetables to set a good example for their children or to prepare healthier food for them [92]. In addition, the observed differences in SRH may also be partly attributable to differences in employment status. Besides the direct effect of employment on income, employment may lead to greater self-esteem, social contacts, and a more structured daily routine [93], which may partially explain the differences in SRH by income. However, employment status also plays an important role in the association between parenthood and health. Mothers are more likely to work part-time than non-mothers, while fathers are more likely to work full-time than non-fathers [94]. Due to reduced working hours, mothers in Germany experience a significant loss of income after the birth of their first child. In the fourth year after giving birth, they earn an average of almost €30,000 less per year than women of the same age without children [95, 96]. Although the associations of income and parental status with SRH decrease after adjusting for education and employment status, some remain significant. Therefore, income and parenthood are partially associated to SRH independently of education and employment status. Hence, further research is needed to better understand the complex associations between income, parental status and SRH, as well as the underlying mechanisms and the impact of societal developments and policy interventions (e.g., family benefits) over time. In the future, the continuous collection of data in panel studies may enable the analysis of the impact of long-term social change on health inequality.

### Limitations and strengths of the analysis

The present analysis has some limitations. One is that the data used for this analysis come from cross-sectional surveys. It is therefore not possible to draw conclusions about the direction of the effects of poverty, parenthood and health (selection versus causality). However, this was not the aim of the study.

Another limitation is that different survey designs were used in 2014/15 and 2024: web-based or written questionnaires, as opposed to telephone interviews in the other GEDA surveys. This change may have affected respondent behaviour [97], given that social desirability has been found to be lower for self-administered surveys without an interviewer [98]. This may have resulted in poorer SRH in 2014/15 and 2024, thereby limiting the comparability of the results over time [97]. However, sensitivity analyses which controlled for survey design produced the same results as those presented here.

When interpreting the results, it should be noted that some groups of the population may be underrepresented, such as people in the low education group, people with limited German language skills or people with health-related limitations [57]. With regard to poverty, this particularly affects groups such as refugees without sufficient German language skills or the homeless. Even weighting procedures cannot compensate for the underrepresentation of these hard-to-reach groups. This may have led to an underestimation of income inequality in health.

SRH is a subjective measure of health, and assessment criteria can vary between different population groups and over time. Therefore, a comparison with more objective health outcomes would be useful in further research. It should be noted that the poverty definition used in this study is based on equivalised household income. This means that household income has been adjusted for household composition. Hence, some of the observed differences may be attributable to weighting for household composition rather than to parental status. However, this is an inherent feature of the concept of relative poverty. As it ensures comparability of different household types and represents the common reference point for relative poverty, this definition is the most appropriate one to apply. Moreover, the at-risk-of-poverty rate is often criticized as a purely one-dimensional measure that focuses solely on income and disregards private wealth [82].

In the present analysis, parenthood is defined by information on household composition. No distinction was made between biological, step, adopted or foster children. The number or age of children in the household was also not addressed in detail. Individuals whose children no longer live in their household are considered non-parents. For separated families, the frequency with which children live in the household was not recorded. Furthermore, while the analysis was adjusted for partner status, no distinction was made between single parents and parents living in partnered households, even though single parents are particularly affected by poverty [82]. Including additional interactions with partner status in the analysis would have been too complex, and the sample sizes, especially for single fathers, would have been too small. Further research is needed to describe the health of single parents in relation to their income situation over time.

Despite the limitations mentioned, the present analysis also has some strengths. The RKI surveys represent large, high-quality datasets that are – with some restrictions – representative of the adult resident population in Germany. Trend analyses benefit from the consistent recording of SRH, income and (with minor changes) household composition over the surveys. This allowed for simultaneous stratification by income groups, parental status and gender, thus enabling the trend analysis of the health status among social subgroups.

### Conclusions

The present analysis provides new insights into the association between income, parenthood and SRH – and how these association have changed between 2009 and 2024: the health of people at risk of poverty living without minor children was already deteriorating in the mid-2010s. But at the end of and after the pandemic, this also applied to mothers and, to a lesser extent, to fathers. This underscores the interweaving of the vertical dimensions (education, occupation position, and income) and horizontal dimensions (parental status, partner status as well as age, gender, ethnicity, region, or occupational groups) of health inequality [99].

The widening income inequality in health to the detriment of people at risk of poverty highlights the need for action. To reduce poverty and improve the health of women and men at risk of poverty, a coordinated package of interventions at the societal and community level is needed in line with the ’Health in All Policies‘ approach [100]. Attention should be paid to the different needs of parents and non-parents.

In order to identify negative health trends in specific population groups at an early stage and to promote and evaluate policies to reduce health inequalities, the interplay of vertical and horizontal dimensions of health inequalities should be monitored more regularly in the future.

## Supplementary Information

Supplementary Material 1.

## Data Availability

The authors state that some access restrictions apply to the data on which the results are based. The dataset cannot be made publicly available, as the informed consent of the study participants does not cover the public provision of the data. The data is accessible for extended use via the Research Data Center (FDZ) of the Robert Koch Institute if there is a justified research interest. Requests can be made by e-mail to fdz@rki.de. Further information can be found on the following website (German): https://www.rki.de/fdz.

## Abbreviations

CASMIN: Comparative Analysis of Social Mobility in Industrial Nations
CATI: Computer Assisted Telephone Interview
COVID-19: Coronavirus disease 2019
EHIS: European Health Interview Survey
EU: European Union
GDP: Gross Domestic Product
GEDA: German Health Update
MEHM: Minimum European Health Module
OECD: Organisation for Economic Co–operation and Development
RII: Relative Index of Inequality
RKI: Robert Koch Institute
SII: Slope Index of Inequality
SRH: Self–rated health
US: United States
95% CI: 95% Confidence Interval
