# Supplementary material for: Poverty and health – does parenthood matter? Trends in income inequality in self-rated health among parents and non-parents in Germany from 2009 to 2024
Source: BMC Public Health. 2026 May 9;26:1518. doi: 10.1186/s12889-026-27495-x (PMC13159269; doi:10.1186/s12889-026-27495-x)
Supplement: Supplementary file 1 — Supplementary Material 1. [file 12889_2026_27495_MOESM1_ESM.docx]

**Appendix to the manuscript:**

**Poverty and health – does parenthood matter? Trends in income inequality in self-rated health among parents and non-parents in Germany from 2009 to 2024**

Petra Rattay, Florian Beese, Stefanie Sperlich, Nico Dragano, Niels Michalski

Tab. A-1 Sample description (total), stratified by gender

Tab. A-2 Income groups stratified by parental status and gender

Tab. A-3 Good SRH among women and men, stratified by survey (age-adjusted prevalence of good/very good SRH as %, 95% CI)

Tab. A-4 Good SRH among women and men, stratified by parental status and survey (age-adjusted prevalence of good/very good SRH as %, 95%-CI)

Tab. A-5 Good SRH among women and men, stratified by income groups and survey (age-adjusted prevalence of good/very good SRH as %, 95%-CI)

Tab. A-6 Adjusted prevalence for good SRH among women and men by income and parental status over time (predicted probabilities of good/very good SRH as %, 95% CI)

Tab. A-7 Absolute and relative income inequalities in good (good/very good) SRH in parents and non-parents, stratified by gender and survey (Slope Index of Inequality (SII), Relative Index of Inequality (RII), 95% CI)

Fig. A-1 Results of the sensitivity analysis: Trends in good SRH among mothers and fathers, stratified by income groups (predicted probabilities of good/very good SRH as %, 95% CI; adjusted for age, partner status, number of children in the household and age of the youngest child)

Tab. A-1 Sample description (total), stratified by gender

|  | **Women** | **Men** |
| --- | --- | --- |
|  | *n (%)^a^* | *n (%)^a^* |
| **Total** | 58,102 | 48,297 |
| **Self-rated health** |  |  |
| Very good / good | 13,329 (26.1) | 9,556 (23.6) |
| Fair / bad / very bad | 44,712 (73.9) | 38,703 (76.4) |
| Missings | 61 | 38 |
| **Age group** |  |  |
| 25-29 | 5,056 (10.7) | 4,693 (11.8) |
| 30-34 | 6,237 (13.2) | 5,547 (13.0) |
| 35-39 | 7,393 (14.2) | 6,105 (14.1) |
| 40-44 | 8,966 (14.7) | 7,378 (14.7) |
| 45-49 | 8,789 (14.9) | 7,361 (14.7) |
| 50-54 | 10,306 (15.4) | 8,358 (15.5) |
| 55-59 | 11,355 (17.0) | 8,855 (16.2) |
| **Income** |  |  |
| <60% (at risk of poverty) | 6,658 (15.9) | 4,435 (13.8) |
| 60%-<150% | 35,099 (62.1) | 26,636 (59.1) |
| >=150% | 16,345 (22.0) | 17,226 (27.1) |
| **Parental status** |  |  |
| Non-parent | 33,780 (58.6) | 30,741 (67.2) |
| Parent | 23,732 (41.4) | 17,008 (32.8) |
| Missings | 590 | 548 |
| **Partner status** |  |  |
| No partner | 17,645 (30.9) | 15,401 (37.2) |
| Partner | 39,803 (69.1) | 32,095 (62.8) |
| Missings | 654 | 801 |
| **Education** |  |  |
| Low | 6,454 (19.6) | 7,203 (25.9) |
| Middle | 31,367 (59.1) | 22,282 (51.6) |
| High | 20,159 (21.3) | 18,692 (22.5) |
| Missings | 122 | 120 |
| **Employment status** |  |  |
| Full-time employed | 23,159 (37.2) | 39,350 (79.3) |
| Part-time employed | 21,662 (36.2) | 2,820 (5.4) |
| Non-employed / Others | 12,644 (26.5) | 5,603 (15.2) |
| Missings | 637 | 524 |

*^a^* n unweighted; % weighted

Tab. A-2 Income groups stratified by parental status and gender

|  |  | **Women** | **Men** |
| --- | --- | --- | --- |
| **Parental status** | **Income** | % (95% CI)^a^ | % (95% CI)^a^ |
| No-parent |  |  |  |
|  | <60% | 15.3 (14.7–15.9) | 14.8 (14.1–15.4) |
|  | 60%-<150% | 58.3 (57.6–59.0) | 54.9 (54.1–55.7) |
|  | =150% | 26.4 (25.8–27.0) | 30.3 (29.7–31.0) |
| Parent |  |  |  |
|  | <60% | 16.1 (15.4–16.8) | 11.2 (10.4–12.0) |
|  | 60%-<150% | 68.1 (67.3–68.9) | 68.3 (67.4–69.3) |
|  | =150% | 15.8 (15.2–16.4) | 20.5 (19.7–21.2) |

*^a^* % weighted

Tab. A-3 Good SRH among women and men, stratified by survey (age-adjusted prevalence of good/very good SRH as %, 95% CI)

|  | **Women** | **Men** |
| --- | --- | --- |
| **Survey** | % (95% CI) | % (95% CI) |
| GEDA09 | 75.7 (74.5–76.9) | 77.7 (76.3–79.2) |
| GEDA10 | 76.4 (75.2–77.5) | 79.4 (78.1–80.8) |
| GEDA12 | 75.2 (73.5–76.8) | 77.1 (75.4–78.7) |
| GEDA14/15-EHIS | 74.5 (73.3–75.7) | 75.6 (74.3–76.9) |
| GEDA19/20-EHIS (before 15.03.) | 74.2 (72.0–76.4) | 74.8 (72.5–77.0) |
| GEDA19/20-EHIS/GEDA21 (from 15.03.) | 80.2 (78.3–82.1) | 81.9 (79.8–83.9) |
| GEDA22 | 74.0 (72.5–75.5) | 75.8 (74.1–77.4) |
| GEDA23 | 70.8 (69.1–72.5) | 75.6 (73.9–77.3) |
| PANEL24 | 68.1 (66.6–69.6) | 71.6 (70.0–73.2) |
| **Total** | 73.1 (72.5–73.6) | 75.6 (75.0–76.1) |

Tab. A-4 Good SRH among women and men, stratified by parental status and survey (age-adjusted prevalence of good/very good SRH as %, 95%-CI)

|  | **Total** | | **Women** | | **Men** | |
| --- | --- | --- | --- | --- | --- | --- |
|  | **Parents** | **No parents** | **Parents** | **No parents** | **Parents** | **No parents** |
| **Survey** | % (95% CI) | % (95% CI) | % (95% CI) | % (95% CI) | % (95% CI) | % (95% CI) |
| GEDA09 | 80.8 (79.1–82.5) | 71.0 (69.2–72.7) | 80.9 (79.1–82.6) | 71.1 (69.4–72.9) | 81.4 (79.1–83.7) | 75.3 (73.4–77.1) |
| GEDA10 | 81.5 (79.9–83.1) | 71.6 (69.9–73.3) | 81.5 (80.0–83.1) | 71.8 (70.1–73.5) | 83.2 (81.2–85.3) | 76.9 (75.1–78.6) |
| GEDA12 | 81.2 (78.8–83.6) | 69.9 (67.7–72.2) | 81.3 (78.9–83.7) | 70.1 (67.8–72.3) | 82.2 (79.6–84.9) | 73.5 (71.4–75.6) |
| GEDA14/15-EHIS | 80.2 (78.4–82.0) | 70.9 (69.4–72.4) | 80.4 (78.6–82.2) | 71.1 (69.6–72.7) | 81.9 (79.8–84.1) | 72.5 (70.9–74.2) |
| GEDA19/20-EHIS (before 15.03.) | 82.2 (79.2–85.2) | 69.4 (66.5–72.4) | 82.2 (79.2–85.2) | 69.4 (66.5–72.4) | 83.3 (80.1–86.6) | 72.2 (69.5–74.9) |
| GEDA19/20-EHIS/GEDA21 (from 15.03.) | 87.7 (85.1–90.3) | 75.3 (72.7–78.0) | 87.8 (85.2–90.4) | 75.4 (72.7–78.0) | 90.5 (88.0–92.9) | 78.4 (75.7–81.1) |
| GEDA22 | 82.1 (80.2–84.1) | 68.3 (66.2–70.5) | 82.1 (80.2–84.1) | 68.5 (66.4–70.6) | 83.9 (81.6–86.2) | 72.7 (70.6–74.7) |
| GEDA23 | 75.4 (72.9–78.0) | 67.6 (65.4–69.9) | 75.5 (72.9–78.0) | 67.8 (65.5–70.0) | 82.6 (79.9–85.3) | 72.7 (70.5–74.8) |
| PANEL24 | 73.2 (70.8–75.7) | 64.4 (62.4–66.3) | 73.3 (70.8–75.8) | 64.5 (62.5–66.5) | 77.1 (74.4–79.7) | 69.7 (67.5–71.8) |
| **Total** | 80.8 (80.3–81.4) | 70.7 (70.2–71.2) | 79.7 (79.0–80.5) | 68.8 (68.1–69.5) | 82.0 (81.2–82.9) | 72.6 (71.9–73.3) |

Tab. A-5 Good SRH among women and men, stratified by income groups and survey (age-adjusted prevalence of good/very good SRH as %, 95%-CI)

|  |  | **Income groups** | | |
| --- | --- | --- | --- | --- |
|  |  | **<60%** | **60%-<150%** | **>=150%** |
|  | **Survey** | % (95% CI) | % (95% CI) | % (95% CI) |
| **Total** |  |  |  |  |
|  | GEDA09 | 64.1 (60.2–67.9) | 75.8 (74.3–77.3) | 83.9 (81.8–86.1) |
|  | GEDA10 | 63.6 (59.9–67.3) | 77.5 (76.0–78.9) | 81.9 (79.6–84.2) |
|  | GEDA12 | 66.6 (61.3–72.0) | 73.6 (71.5–75.7) | 83.8 (81.4–86.2) |
|  | GEDA14/15-EHIS | 61.8 (58.5–65.2) | 75.3 (73.8–76.7) | 81.0 (78.9–83.1) |
|  | GEDA19/20-EHIS (before 15.03.) | 52.7 (46.0–59.4) | 76.4 (73.8–79.0) | 85.8 (82.9–88.8) |
|  | GEDA19/20-EHIS/GEDA21 (from 15.03.) | 65.2 (59.1–71.3) | 81.0 (78.5–83.5) | 88.9 (86.3–91.4) |
|  | GEDA22 | 53.2 (48.3–58.0) | 76.2 (74.3–78.0) | 82.6 (80.2–84.9) |
|  | GEDA23 | 51.7 (46.5–56.9) | 70.9 (68.8–73.0) | 84.4 (82.2–86.7) |
|  | PANEL24 | 52.7 (47.8–57.5) | 68.6 (66.7–70.5) | 75.7 (73.3–78.0) |
|  | **Total** | 58.3 (57.0–59.6) | 74.8 (74.3–75.3) | 83.2 (82.6–83.8) |
| **Women** |  |  |  |  |
|  | GEDA09 | 64.2 (60.3–68.0) | 75.9 (74.4–77.4) | 84.0 (81.8–86.1) |
|  | GEDA10 | 63.9 (60.2–67.6) | 77.5 (76.1–78.9) | 82.0 (79.7–84.3) |
|  | GEDA12 | 67.0 (61.7–72.3) | 73.7 (71.6–75.8) | 83.8 (81.4–86.2) |
|  | GEDA14/15-EHIS | 62.1 (58.7–65.4) | 75.4 (74.0–76.9) | 81.3 (79.2–83.4) |
|  | GEDA19/20-EHIS (before 15.03.) | 52.7 (46.0–59.4) | 76.4 (73.8–79.0) | 85.8 (82.9–88.8) |
|  | GEDA19/20-EHIS/GEDA21 (from 15.03.) | 65.2 (59.1–71.3) | 81.1 (78.6–83.6) | 88.9 (86.4–91.4) |
|  | GEDA22 | 53.5 (48.7–58.4) | 76.2 (74.4–78.0) | 82.6 (80.2–84.9) |
|  | GEDA23 | 52.1 (46.9–57.3) | 71.0 (68.9–73.1) | 84.4 (82.2–86.7) |
|  | PANEL24 | 53.3 (48.4–58.1) | 68.7 (66.8–70.6) | 75.7 (73.3–78.1) |
|  | **Total** | 57.8 (56.1–59.4) | 74.0 (73.4–74.6) | 81.8 (81.0–82.7) |
| **Men** |  |  |  |  |
|  | GEDA09 | 66.3 (60.8–71.8) | 77.2 (75.4–79.0) | 84.9 (82.7–87.0) |
|  | GEDA10 | 64.8 (59.7–70.0) | 79.1 (77.4–80.8) | 87.5 (85.6–89.5) |
|  | GEDA12 | 67.0 (61.2–72.8) | 76.9 (74.7–79.1) | 81.4 (78.8–84.0) |
|  | GEDA14/15-EHIS | 55.5 (51.1–59.9) | 75.7 (74.0–77.3) | 84.8 (82.8–86.9) |
|  | GEDA19/20-EHIS (before 15.03.) | 53.5 (47.0–60.0) | 75.4 (72.5–78.3) | 88.2 (85.4–91.0) |
|  | GEDA19/20-EHIS/GEDA21 (from 15.03.) | 61.9 (54.6–69.2) | 83.3 (80.7–86.0) | 90.5 (88.4–92.6) |
|  | GEDA22 | 58.1 (52.3–63.9) | 75.6 (73.5–77.8) | 84.9 (82.7–87.2) |
|  | GEDA23 | 61.8 (56.3–67.4) | 74.8 (72.6–77.1) | 85.6 (83.3–87.9) |
|  | PANEL24 | 51.4 (46.1–56.7) | 71.5 (69.3–73.7) | 80.7 (78.3–83.1) |
|  | **Total** | 59.1 (57.2–61.1) | 75.7 (75.0–76.4) | 84.3 (83.5–85.1) |

Tab. A-6 Adjusted prevalence for good SRH among women and men by income and parental status over time (predicted probabilities of good/very good SRH as %, 95% CI)

|  |  |  | **Women** | | **Men** | |
| --- | --- | --- | --- | --- | --- | --- |
|  |  |  | **Model 1^a^** | **Model 2^b^** | **Model 1^a^** | **Model 2^b^** |
| **Survey** | **Parental status** | **Income** | % (95% CI) | % (95% CI) | % (95% CI) | % (95% CI) |
| 2009 | Non-parent | <60% | 58.5 (52.4–64.5) | 64.5(57.8–71.1) | 64.8 (58.0–71.6) | 77.4(69.5–85.4) |
| 2009 | Non-parent | 60%-<150% | 72.3 (70.0–74.6) | 73.8(71.5–76.1) | 73.6 (71.1–76.1) | 75.5(73.0–78.0) |
| 2009 | Non-parent | =150% | 82.3 (79.3–85.4) | 78.4(75.4–81.4) | 83.3 (80.6–86.0) | 79.3(76.7–81.8) |
| 2009 | Parent | <60% | 67.8 (62.6–72.9) | 77.1(71.3–82.8) | 63.1 (54.2–72.0) | 70.5(60.7–80.4) |
| 2009 | Parent | 60%-<150% | 77.3 (75.3–79.3) | 81.0(79.0–83.1) | 78.2 (75.6–80.7) | 78.5(76.1–81.0) |
| 2009 | Parent | =150% | 85.5 (82.5–88.4) | 83.7(80.8–86.6) | 85.6 (82.0–89.3) | 80.1(76.8–83.4) |
| 2010 | Non-parent | <60% | 57.4 (51.6–63.2) | 64.2(57.8–70.7) | 61.1 (54.8–67.4) | 74.1(66.8–81.5) |
| 2010 | Non-parent | 60%-<150% | 73.1 (70.8–75.4) | 74.4(72.1–76.6) | 75.5 (73.0–78.0) | 77.1(74.6–79.6) |
| 2010 | Non-parent | =150% | 81.3 (78.3–84.2) | 77.6(74.8–80.3) | 85.9 (83.4–88.4) | 81.8(79.5–84.1) |
| 2010 | Parent | <60% | 67.0 (62.1–71.9) | 75.6(70.0–81.1) | 63.1 (54.6–71.7) | 73.6(64.0–83.2) |
| 2010 | Parent | 60%-<150% | 79.5 (77.8–81.3) | 81.4(79.6–83.2) | 80.1 (77.8–82.3) | 79.9(77.6–82.1) |
| 2010 | Parent | =150% | 82.3 (78.4–86.1) | 80.6(77.0–84.2) | 90.9 (88.4–93.4) | 83.8(81.4–86.2) |
| 2012 | Non-parent | <60% | 65.3 (57.8–72.7) | 70.9(62.2–79.6) | 66.1 (59.3–72.9) | 76.2(69.3–83.1) |
| 2012 | Non-parent | 60%-<150% | 68.0 (64.7–71.3) | 69.1(65.8–72.3) | 72.0 (69.0–75.1) | 74.3(71.3–77.4) |
| 2012 | Non-parent | =150% | 83.3 (80.1–86.4) | 78.6(75.6–81.6) | 80.1 (76.8–83.3) | 77.0(73.9–80.1) |
| 2012 | Parent | <60% | 66.9 (59.5–74.3) | 74.6(66.8–82.4) | 64.6 (54.8–74.4) | 69.9(59.8–79.9) |
| 2012 | Parent | 60%-<150% | 78.8 (76.1–81.5) | 80.2(77.4–82.9) | 78.7 (75.5–81.8) | 78.1(75.2–81.0) |
| 2012 | Parent | =150% | 86.1 (82.6–89.6) | 82.7(79.3–86.1) | 88.4 (84.4–92.5) | 81.8(78.1–85.5) |
| 2014/15 | Non-parent | <60% | 54.4 (49.6–59.2) | 59.6(54.4–64.7) | 50.4 (45.0–55.9) | 60.5(54.0–66.9) |
| 2014/15 | Non-parent | 60%-<150% | 73.3 (71.3–75.4) | 72.8(70.8–74.9) | 72.5 (70.3–74.7) | 72.5(70.3–74.7) |
| 2014/15 | Non-parent | =150% | 80.5 (77.9–83.0) | 75.5(73.0–77.9) | 83.0 (80.4–85.6) | 78.1(75.7–80.6) |
| 2014/15 | Parent | <60% | 71.6 (66.8–76.5) | 78.2(72.8–83.7) | 61.3 (52.5–70.1) | 66.1(57.1–75.2) |
| 2014/15 | Parent | 60%-<150% | 77.4 (75.3–79.5) | 79.2(77.0–81.4) | 78.7 (76.2–81.3) | 77.0(74.6–79.5) |
| 2014/15 | Parent | =150% | 83.0 (79.2–86.7) | 80.3(76.7–83.9) | 89.1 (86.2–91.9) | 81.3(78.7–83.9) |
| 2019/20 (a) | Non-parent | <60% | 42.9 (34.2–51.6) | 47.9(38.7–57.1) | 52.6 (44.6–60.6) | 63.2(53.5–73.0) |
| 2019/20 (a) | Non-parent | 60%-<150% | 72.8 (68.8–76.8) | 71.5(67.7–75.2) | 74.7 (70.9–78.5) | 75.3(71.7–79.0) |
| 2019/20 (a) | Non-parent | =150% | 88.6 (85.2–91.9) | 81.3(78.1–84.5) | 89.0 (85.5–92.6) | 82.1(78.9–85.4) |
| 2019/20 (a) | Parent | <60% | 67.3 (57.0–77.6) | 74.1(63.0–85.1) | 68.9 (56.5–81.4) | 75.4(61.6–89.3) |
| 2019/20 (a) | Parent | 60%-<150% | 81.8 (78.8–84.8) | 80.9(77.9–83.9) | 79.4 (75.3–83.4) | 77.7(73.8–81.7) |
| 2019/20 (a) | Parent | =150% | 84.0 (78.0–90.0) | 78.4(72.8–84.0) | 92.5 (88.7–96.4) | 84.5(81.0–88.1) |
| 2020/21 (b) | Non-parent | <60% | 57.7 (49.2–66.2) | 63.2(54.1–72.4) | 59.8 (51.0–68.7) | 75.8(65.1–86.6) |
| 2020/21 (b) | Non-parent | 60%-<150% | 78.7 (74.9–82.5) | 77.5(74.0–80.9) | 80.7 (77.0–84.4) | 81.0(77.3–84.6) |
| 2020/21 (b) | Non-parent | =150% | 88.4 (85.0–91.9) | 81.2(78.0–84.3) | 91.3 (88.5–94.2) | 83.7(80.9–86.6) |
| 2020/21 (b) | Parent | <60% | 79.7 (71.2–88.1) | 88.8(78.9–98.7) | 73.0 (60.9–85.2) | 77.7(63.7–91.6) |
| 2020/21 (b) | Parent | 60%-<150% | 84.7 (81.6–87.9) | 85.5(82.3–88.7) | 89.3 (86.6–92.0) | 87.7(85.0–90.4) |
| 2020/21 (b) | Parent | =150% | 90.2 (87.1–93.3) | 83.2(80.2–86.2) | 94.0 (91.7–96.4) | 86.9(84.3–89.5) |
| 2022 | Non-parent | <60% | 44.8 (38.4–51.2) | 49.2(42.3–56.1) | 60.3 (53.5–67.2) | 74.4(66.1–82.8) |
| 2022 | Non-parent | 60%-<150% | 73.5 (70.6–76.3) | 71.8(69.0–74.5) | 73.1 (70.2–76.1) | 73.0(70.2–75.9) |
| 2022 | Non-parent | =150% | 82.0 (78.8–85.2) | 75.8(72.9–78.8) | 86.7 (83.8–89.6) | 79.8(77.1–82.4) |
| 2022 | Parent | <60% | 71.1 (64.2–78.0) | 77.6(69.9–85.3) | 62.8 (50.5–75.1) | 71.3(57.4–85.1) |
| 2022 | Parent | 60%-<150% | 80.5 (78.2–82.9) | 80.1(77.6–82.5) | 83.1 (80.5–85.8) | 80.5(78.0–83.0) |
| 2022 | Parent | =150% | 85.3 (82.0–88.6) | 79.2(76.1–82.3) | 84.5 (80.9–88.0) | 77.8(74.6–81.0) |
| 2023 | Non-parent | <60% | 50.3 (43.3–57.3) | 55.9(48.1–63.6) | 57.5 (50.7–64.3) | 72.5(63.9–81.1) |
| 2023 | Non-parent | 60%-<150% | 68.6 (65.4–71.8) | 66.8(63.7–69.8) | 74.0 (71.0–77.1) | 73.4(70.4–76.4) |
| 2023 | Non-parent | =150% | 84.8 (81.9–87.7) | 77.5(74.7–80.3) | 85.9 (83.1–88.7) | 79.7(77.1–82.3) |
| 2023 | Parent | <60% | 55.9 (47.4–64.4) | 62.0(52.7–71.3) | 74.1 (64.3–83.9) | 79.8(69.0–90.5) |
| 2023 | Parent | 60%-<150% | 75.3 (72.5–78.1) | 74.4(71.6–77.1) | 79.2 (75.8–82.6) | 76.8(73.7–79.9) |
| 2023 | Parent | =150% | 85.0 (81.4–88.7) | 78.8(75.5–82.1) | 91.5 (88.5–94.4) | 83.8(80.8–86.8) |
| 2024 | Non-parent | <60% | 46.2 (39.6–52.8) | 50.7(43.6–57.8) | 46.7 (39.7–53.7) | 58.1(49.6–66.6) |
| 2024 | Non-parent | 60%-<150% | 65.6 (62.8–68.4) | 64.4(61.7–67.1) | 68.5 (65.4–71.6) | 69.0(66.0–72.0) |
| 2024 | Non-parent | =150% | 73.9 (70.9–76.9) | 68.5(65.7–71.2) | 80.1 (77.2–82.9) | 74.0(71.4–76.5) |
| 2024 | Parent | <60% | 56.7 (49.0–64.5) | 61.7(53.6–69.8) | 57.0 (47.4–66.6) | 63.2(53.2–73.2) |
| 2024 | Parent | 60%-<150% | 71.8 (69.0–74.6) | 71.4(68.6–74.1) | 75.4 (72.3–78.5) | 73.6(70.6–76.5) |
| 2024 | Parent | =150% | 80.0 (75.9–84.0) | 74.5(70.9–78.1) | 84.2 (79.7–88.7) | 77.0(73.1–80.9) |

^a^ Model 1: adjusted for age groups and partner status

^b^ Model 2: adjusted for age groups, partner status, education and employment status

Tab. A-7 Absolute and relative income inequalities in good (good/very good) SRH in parents and non-parents, stratified by gender and survey (Slope Index of Inequality (SII), Relative Index of Inequality (RII), 95% CI, adjusted for age groups and partner status)

|  |  | **Women** | **Men** | **Women** | **Men** |
| --- | --- | --- | --- | --- | --- |
| **Survey** | **Parental status** | **SII (95% CI)** | **SII (95% CI)** | **RII (95% CI)** | **RII (95% CI)** |
| GEDA09 | Non-parent | 0.27 (0.20–0.35) | 0.22 (0.15–0.29) | 1.47 (1.33–1.64) | 1.34 (1.22–1.48) |
| GEDA10 | Non-parent | 0.27 (0.20–0.34) | 0.29 (0.22–0.36) | 1.46 (1.32–1.61) | 1.46 (1.33–1.60) |
| GEDA12 | Non-parent | 0.26 (0.17–0.34) | 0.20 (0.12–0.29) | 1.44 (1.27–1.63) | 1.32 (1.18–1.48) |
| GEDA14/15-EHIS | Non-parent | 0.29 (0.23–0.35) | 0.37 (0.30–0.44) | 1.51 (1.38–1.65) | 1.68 (1.52–1.85) |
| GEDA19/20-EHIS (before 15.03.) | Non-parent | 0.53 (0.42–0.64) | 0.42 (0.32–0.52) | 2.18 (1.81–2.61) | 1.81 (1.55–2.12) |
| GEDA19/20-EHIS/GEDA21 (from 15.03.) | Non-parent | 0.37 (0.27–0.48) | 0.34 (0.24–0.44) | 1.65 (1.42–1.92) | 1.55 (1.35–1.79) |
| GEDA22 | Non-parent | 0.42 (0.34–0.50) | 0.30 (0.22–0.39) | 1.86 (1.64–2.12) | 1.53 (1.36–1.72) |
| GEDA23 | Non-parent | 0.41 (0.32–0.49) | 0.32 (0.24–0.41) | 1.83 (1.59–2.10) | 1.57 (1.39–1.77) |
| PANEL24 | Non-parent | 0.30 (0.22–0.38) | 0.38 (0.30–0.46) | 1.60 (1.40–1.82) | 1.74 (1.53–1.97) |
| GEDA09 | Parent | 0.20 (0.13–0.28) | 0.27 (0.15–0.38) | 1.29 (1.17–1.41) | 1.39 (1.20–1.60) |
| GEDA10 | Parent | 0.19 (0.11–0.27) | 0.33 (0.23–0.44) | 1.26 (1.14–1.40) | 1.49 (1.31–1.69) |
| GEDA12 | Parent | 0.25 (0.14–0.35) | 0.28 (0.17–0.40) | 1.35 (1.18–1.54) | 1.41 (1.22–1.63) |
| GEDA14/15-EHIS | Parent | 0.13 (0.05–0.21) | 0.32 (0.23–0.42) | 1.18 (1.07–1.30) | 1.48 (1.32–1.67) |
| GEDA19/20-EHIS (before 15.03.) | Parent | 0.21 (0.07–0.36) | 0.31 (0.17–0.45) | 1.30 (1.08–1.57) | 1.45 (1.22–1.72) |
| GEDA19/20-EHIS/GEDA21 (from 15.03.) | Parent | 0.10 (0.00–0.21) | 0.22 (0.09–0.34) | 1.13 (1.00–1.27) | 1.27 (1.10–1.47) |
| GEDA22 | Parent | 0.15 (0.06–0.24) | 0.17 (0.06–0.29) | 1.20 (1.07–1.34) | 1.23 (1.07–1.42) |
| GEDA23 | Parent | 0.34 (0.22–0.46) | 0.22 (0.11–0.33) | 1.56 (1.32–1.84) | 1.30 (1.14–1.49) |
| PANEL24 | Parent | 0.29 (0.18–0.40) | 0.33 (0.20–0.45) | 1.48 (1.27–1.73) | 1.52 (1.30–1.79) |


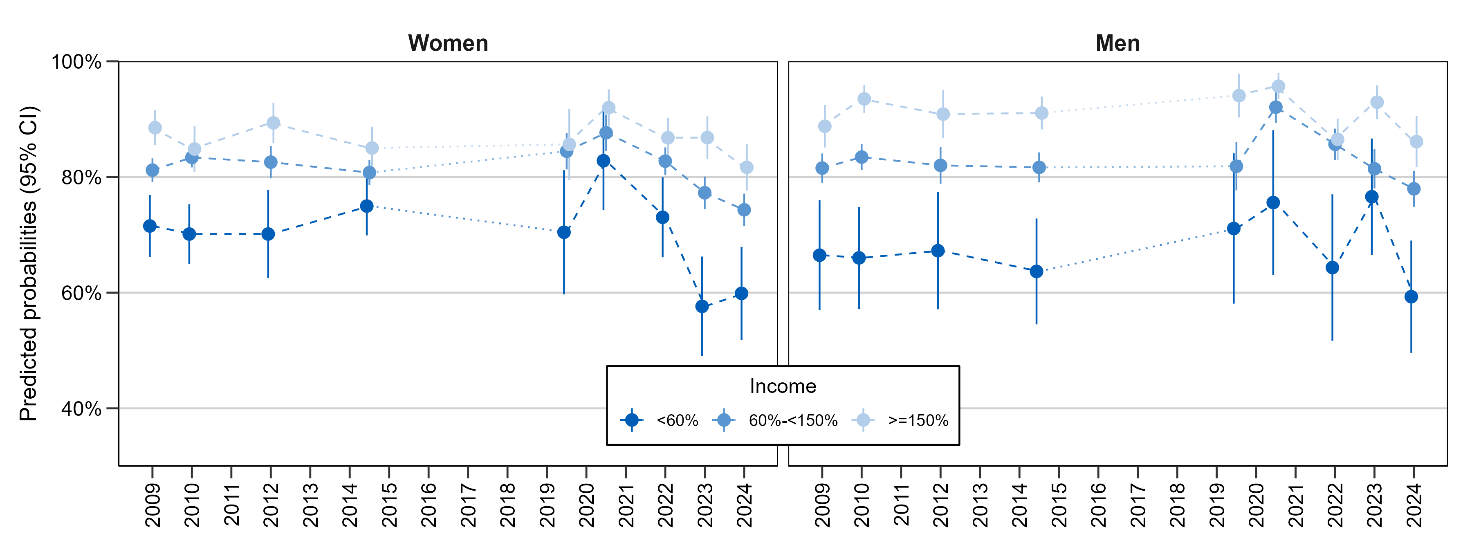
Fig. A-1 Results of the sensitivity analysis: Trends in good SRH among mothers and fathers, stratified by income groups (predicted probabilities of good/very good SRH as %, 95% CI; adjusted for age groups, partner status, number of children in the household and age of the youngest child)

Legend: Age-adjusted prevalence rates from repeated cross-sectional surveys. Each point represents a separate sample; lines indicate trends over time.
